# Cosmic hylomorphism: A powerist ontology of quantum mechanics

**DOI:** 10.1007/s13194-020-00342-5

**Published:** 2021-01-15

**Authors:** William M. R. Simpson

**Affiliations:** Wolfson College, University of Cambridge, Cambridge, UK

**Keywords:** Primitive ontology, Quantum mechanics, Powers, Hylomorphism

## Abstract

The primitive ontology approach to quantum mechanics seeks to account for quantum phenomena in terms of a distribution of matter in three-dimensional space (or four-dimensional spacetime) and a law of nature that describes its temporal development. This approach to explaining quantum phenomena is compatible with either a Humean or powerist account of laws. In this paper, I offer a powerist ontology in which the law is specified by Bohmian mechanics for a global configuration of particles. Unlike in other powerist ontologies, however, this law is not grounded in a *structural power* that is instantiated by the global configuration. Instead, I combine the primitive ontology approach with Aristotle’s doctrine of hylomorphism to create a new metaphysical model, in which the cosmos is a hylomorphic substance with an *intrinsic power* to choreograph the trajectories of the particles.

## Introduction

The primitive ontology approach to quantum mechanics seeks to provide an ontology of nature that supports a realist interpretation of quantum mechanics and accommodates phenomena like quantum entanglement (Allori et al. 2008, 2014; Esfeld 2014a). One of the challenges facing any realist interpretation is the problem of specifying the ontological status of the wave function that determines the quantal properties of a physical system. The wave function of a system of *N* particles is not defined in a three-dimensional physical space, but in an abstract 3*N*-dimensional configuration space. According to the primitive ontology approach, the wave function is not part of the primitive ontology, but enters the account through the nomological role it plays in specifying the dynamics of a primitive distribution of matter in three-dimensional space (or four-dimensional spacetime).

Maudlin has argued that the choice of dynamics comes down to two alternatives (Maudlin 1995) (cf. Bell 1987): either we should adopt something like the modified GRW version of the Schrödinger equation proposed by Ghirardi et al. (1986), in which the wave function is permitted to undergo spontaneous collapse, or something like the pilot wave theory of de Broglie (1928) and Bohm 1951, 1952a, b), which includes an equation of motion for a particle configuration. In the GRWm primitive ontology, for example, there is a gunky distribution of matter, and the law of nature describes the spread and spontaneous contraction in its density (Allori et al. 2008). This model relies upon the GRW modification of quantum mechanics. By contrast, in the Bohmian primitive ontology (Goldstein et al. 2005a, b), the matter consists of particles (or atoms) that have well-defined positions, and the law of nature describes their trajectories. This model relies upon a particular version of the pilot wave theory that has been championed by Dürr, Goldstein and Zanghí under the name of ‘Bohmian mechanics’ since the 1990s (Dürr et al. 1992, 1997).^1^

The matter in both of these models is ‘primitive’ in three senses: first, it cannot be read off the formalism of standard quantum mechanics, or deduced from any other concept or the vocabulary of a given theory, but is posited for the sake of empirical adequacy; secondly, it has ‘no intrinsic physical properties at all’ (Esfeld et al. 2017, p.135) (or, at least, no intrinsic sparse natural properties that can be picked out by our best physical theories); thirdly, every physical phenomenon is ontologically explained and reduced to the motion of the primitive matter. A primitive ontology approach to quantum mechanics aims to explain the measurement outcomes of quantum experiments, like the famous EPR experiment (Einstein et al. 1935), and more generally to explain the behaviour of the macroscopic objects upon which scientists depend to make their measurements, by offering an account of the empirical content of a physical theory that is exhausted by its statements about this primitive distribution of matter (Maudlin 2019).

There are two ways of spelling out the nomological role of the wave function in the temporal development of the matter: namely, by appealing to a Humean account of laws, in which laws describe regularities, or to some form of powerism, in which laws are grounded in powers (Esfeld et al. 2017). In this paper, I will develop a powerist version of the Bohmian primitive ontology, which offers explanatory advantages over existing proposals. In Section 2, I discuss the motivations for offering a powerist ontology in which the power that grounds the law of motion is not a *structural power* instantiated by the particle configuration, but an *intrinsic* power of the cosmos as a whole. In Section 3, I explain how a Bohmian cosmos can have an intrinsic and powerful nature, by introducing a ‘Cosmic Form’ into the fundamental ontology, in addition to the primitive matter, which unites itself to the particle configuration by metaphysically grounding their causal powers. In Section 4, I consider how to secure the explanatory advantage that I have claimed for this metaphysical model against the charge that, in order to explain the phenomenon of quantum entanglement, this power would have to operate in an implausible way.

## In search of Bohmian powers

According to scientific realists, scientific inquiry is our best way of finding out the properties of nature. Recent work in the metaphysics of science has been marked by a resurgent interest in the relationship between the properties that exist in nature and the powers they confer upon physical entities. Contemporary metaphysicians can be divided into *powerists* (or dispositionalists) and *Humeans* (and semi-Humeans)^2^ according to whether they consider these powers to be conferred contingently or necessarily. For Humeans, the relationship between properties and their powers is entirely contingent. Any distribution of perfectly natural properties is possible since there are no necessary connections in nature. For powerists, by contrast, the causal powers conferred by a basic property are intrinsic to the nature of that property itself, or basic properties are to be identified with causal powers. Consequently, it is not possible that the powers of two fundamental properties could ever be swapped.

According to the primitive ontology approach to quantum mechanics, however, the matter which composes the objects of scientific inquiry lacks any intrinsic physical properties. Nonetheless, the wave function is supposed to play a ‘nomological’ role in the temporal development of the matter, and this role requires further elaboration (Esfeld et al. 2017). To better characterise this project, it is helpful to adopt the distinction proposed by Allori et al. between the *primitive ontology* of a physical theory, which concerns exclusively the distribution of matter in physical space, and its *ontology* (Allori et al. 2008, 2014), which encompass all of the other mathematical entities, structures, properties and laws, etc., that may be needed in addition to the primitive ontology to build a coherent metaphysics of physics. In this paper, I wish to propose a new ontology for Bohmian mechanics, rather than suggest a new primitive ontology *tout court*.

Esfeld has recently proposed an ontology for Bohmian mechanics that combines the primitive ontology approach to quantum mechanics with ontic structural realism and adopts a Humean ‘best systems’ account of laws (Esfeld and Deckert 2017). According to Esfeld, the world is composed of ‘matter points’, which are holistically individuated by the distance relations in which they stand. They have no *intrinsic* physical properties or hidden quiddities, only the *structural* property of position conferred by their distance relations. To accommodate the truth of the Bohmian laws, Esfeld adopts the Mill-Ramsey-Lewis account, in which a regularity only qualifies as a law of nature just in case it is an axiom in the ‘best system’ that balances strength and simplicity in deriving the facts about the positions of the matter points. This ‘Super-Humean’ metaphysics of physics is elegantly simple and offers the most developed primitive ontology approach to quantum mechanics that is available at present (Esfeld and Deckert 2017).

However, many philosophers have found Humean accounts of laws to be deeply unsatisfactory. According to Humeans, the Bohmian laws that physicists have put forward, in order to explain such phenomena as quantum entanglement, depend for their *lawfulness* upon the global configuration of particles, and are thus constituted by that which they seek to explain. Yet, as Armstrong complained, ‘a fact cannot be used to explain itself’ (Armstrong 1983, p.40). In making this claim, Armstrong was echoing Plato’s insight that things are explained by being referred to a ‘higher principle’. The Mill-Ramsey-Lewis account, however, not only fails to maintain the necessary metaphysical distance between *explanans* and *explanandum*, but reverses the proper order of explanation: laws are supposed to explain instances that fall under them, yet the lawfulness of laws in this model is grounded in the instances they are supposed to explain.

Doubts have also been raised about the logical coherence of the Super-Humean attempt to make the doctrine of Humean Supervenience compatible with the phenomenon of quantum entanglement (Miller 2013; Esfeld 2014b). For example, in rejecting the standard neo-Humean commitment to an ontology of sparse natural properties, the primitive ontology approach adopted by Super-Humeans removes an objective constraint upon rival systems of laws, leading to subjectivism about the laws of nature (Matarese 2018). It has also been called into question whether Super-Humean matter points can serve as the referents of the Bohmian law of motion, since there is good reason to doubt that matter points persist through time (Simpson 2020). (For further criticisms, see Lazarovici (2018) and Wilson (2018).)

In the alternative ontology I wish to propose, which inherits the primitive ontology of Goldstein et al. (2005a, b), I shall favour an Aristotelian-essentialist account of laws, in which laws neither govern nor describe the physical world, but express the nature of powers (Bird 2007, chp. 9). Powerist ontologies for quantum mechanics have received less attention than Humean ontologies. In order to build a metaphysical model that incorporates a power to choreograph the trajectories of the particles according to the Bohmian law of motion, however, a powerist must answer a number of metaphysical questions. To begin with, what sort of thing might be supposed to *possess* a causal power which could ground the Bohmian law of motion? There are two answers one might be tempted to give. First, we might posit the existence of an entity, in *addition* to the particles that compose the objects of scientific inquiry, which has an intrinsic power to move all of the particles through physical space. Secondly, we might deny that this power is manifested by some separate agent that transcends the world of physical particles, and seek to explain how the cosmos *as a whole* may be said to have an immanent power to govern their trajectories.

### A transcendent power

Let us consider the first option. Suppose there is some substance that exists in addition to the particles, which explains the change in the velocities of the particles by being the *efficient cause* of their motion through space. In that case, the power to choreograph the trajectories of the particles would be an active power of this substance, and the particles would have passive powers to have their velocities changed. Such an account, however, runs into an *interaction dilemma*, which can be generated by the question: does this substance exist in physical space, or does it transcend physical space?

On the one hand, suppose that this substance transcends physical space. In that case, we might consider adopting a dualist approach to quantum mechanics, combining a primitive ontology of Bohmian particles that exist in a low-dimensional physical space with the existence of an entity characterised by the wave function that exists in a high-dimensional configuration space. However, if the substance is a transcendent entity that does not exist in physical space, how can it be the *efficient cause* of the motions of particles in physical space? In such a model, there could be no physical contact between the substance and the particles, and hence no physical conditions for the exercise of its causal power. Barring a fundamental randomness in the exercise of powers, which would be inconsistent with a deterministic Bohmian picture of nature, such a substance would have to exercise its power necessarily rather than contingently, and its causal influence would have to reach between two disparate realms (Esfeld et al. 2017, pp.133-34). Any active powers to bring about change would belong to this transcendent substance, and would thus exist beyond physical space, whilst the world of physical particles would merely be passive and epiphenomenal, its particles reduced to being a decoration of the wave function (Brown and Wallace 2005). We might be tempted to apply Ockham’s razor, in that case, and remove them from the primitive ontology.

On the other hand, suppose this substance exists in physical space. In the context of the Bohmian primitive ontology, such a substance would have to stand in distance relations to the particles. In that case, we face a second sub-dilemma. Either this substance is a local entity (it exists *somewhere* in space) or it is a global entity (it exists *everywhere* in space). If the substance is localised in space (where, in particular?), then it must be at a greater distance from some particles than from others. In that case, the substance must defy the superluminal ban on signalling in modern physics, by exercising its causal power to choreograph the trajectories of all of the particles simultaneously. I shall rule out any such possibility for the purpose of this discussion. If the substance is globalised in space, however, we might think of it as being a field-like entity that is simultaneously in contact with all of the particles in space (Hubert and Romano 2018), such that for every particle there is some part of the substance that is zero distance from that particle. In that case, the substance would not require a superluminal mechanism, nor need it manifest its power necessarily: the condition of it exercising its power is that it is in physical contact with all of the particles. This seems like a better option.

Nonetheless, if the particles that compose the objects of scientific inquiry are endowed merely with *passive* powers to have their velocities changed by a global substance, which is not itself composed of particles, in what sense do causal relations have any place in the world of scientific inquiry? Granted, this global substance is located in physical space. Yet why should we treat the particles that compose the various systems that scientists study as if they were concrete and separate entities that exist over and above this global substance? Once again, we might simply apply Ockham’s razor and treat these particles as epiphenomenal instead. Since it is a guiding assumption of the primitive ontology approach to quantum mechanics that the objects of scientific inquiry are real and their parts are localised within physical space, I shall set aside this possibility as well.

### An immanent power

The second option is the one I should like to consider further in this paper: namely, the possibility that the cosmos itself, inclusive of all of the particles, has a non-local yet immanent power to choreograph their trajectories through physical space. According to Dorato and Esfeld, such a power would have to be a *structural* power instantiated by the global particle configuration, rather than an intrinsic power of any of the objects of scientific inquiry: ‘when it comes to quantum mechanics... it is no longer possible to maintain that the laws are grounded in local or intrinsic properties of particles’ (Dorato and Esfeld 2015, p.420). Yet such an account runs into an *instantiation dilemma*: is this structural power instantiated by the global configuration in virtue of the properties of the particles, or is it instantiated simply as a brute matter of fact?

On the one hand, suppose this structural power is instantiated by the global configuration of particles in virtue of their intrinsic natures. What sort of nature, in that case, would each of the particles require, in order for the configuration *as a whole* to instantiate a structural power? In a primitive ontology approach to quantum mechanics, such properties could not be sparse natural properties that are picked out by our best physics, but would have to be properties that are predicated purely for the sake of explanation. Allowing for this possibility, suppose each of the particles has a multi-track power to change their velocity such that each spatial configuration stimulates a different track of this multi-track power. In that case, we might think of the particle configuration as instantiating a structural power, in virtue of the role that each spatial configuration plays as a whole in stimulating each multi-track power, although it would be the intrinsic powers of each of the particles that ultimately explain the motion of the configuration as a whole. Such a model, however, suggests an implausibly elaborate conspiracy among microscopic particles in nature, in the absence of the existence of anything over and above the particles to coordinate their motions. It also comes at the cost of an extremely abundant ontology of fundamental powers; a different multi-track power for each particle in the cosmos, no less.

On the other hand, suppose this structural power is *not* instantiated in virtue of the intrinsic natures of the particles composing the configuration, but is instantiated as a brute matter of fact. Such a move comes at a theoretical cost: why should we require this entity to be ontologically dependent upon the particles, since the wave function does not supervene upon the particles, rather than a separate element of the primitive ontology with its own causal powers? To do so looks like an *ad hoc* move to avoid the interaction problem. Moreover, how would positing this structural power constitute a scientifically *informative* explanation of their behaviour that can avoid Molière-type objections? To say that an object of scientific inquiry *S* has a power to cause a certain manifestation *M* is to predicate a property *ϕ* of *S* in virtue of which *S* produces *M* in the right circumstances. As Chakravartty notes, this is potentially a scientifically informative claim in the context of an inquiry in which it is reasonable to assume that the power *ϕ* might not have been *intrinsic* to *S* (such that *S* might reasonably be expected to manifest *M* in another possible world), but due to some *extrinsic* feature of its environment (such that a duplicate of *S* might lack *ϕ*) (Chakravartty 2007, pp.125-6). However, the sort of power under consideration is not intrinsic to any of the objects of scientific inquiry, nor does it depend for its efficacy upon their intrinsic natures. It is doubtful, then, whether appealing to such an entity offers any advance on Super-Humeanism in *explaining* how the particles are distributed in space and time. Why shouldn’t a duplicate of the particle configuration instantiate a different structural power?

Yet there may be a third way. A powerist might propose that the plurality of particles has a single, collective property – namely, the property of instantiating a power to choreograph their trajectories – that is *intrinsic* to the plurality of particles, but not to any of the individual particles. Would such a configuration count as an object of scientific inquiry with its own nature? On the one hand, we might choose to withhold any singular variable to stand for the cosmos as a whole, and use a Boolosian plural variable instead. After all, Ockham’s razor rules against postulating entities beyond necessity. In that case, the cosmos would not exist as a singular entity in a Quinean sense. On the other hand, we might be tempted to extend the domain of singular quantification to include all pluralities with fundamentally collective causal powers. After all, if a plurality has an irreducibly collective, metaphysically primitive causal power, then surely that gives us a reason to count this plurality as a substance in the relevant sense (Merricks 2001). In that case, we might say that the cosmos ‘exists’, in addition to the particles, in an extended sense. What we would still require, however, in order to gain an explanatory advantage over Super-Humeanism, is a metaphysical model that can explain the diachronic and transworld sameness of the collective causal power of this particle configuration. This is what I hope to provide in the discussion that follows.

## Cosmic hylomorphism proposed

My new metaphysical model combines the primitive ontology of Bohmian mechanics put forward by Goldstein et al. (2005a, b) with a contemporary adaptation of Aristotle’s hylomorphic theory of substances proposed by Koons (2018, 2014), to produce a powerist account of the temporal development of the primitive distribution of matter. The core of my model, which I shall call *Cosmic Hylomorphism*, may be laid out in four axioms:

1. There are ‘Power-Atoms’, which exercise causal powers to change their velocities in response to their spatial configuration.
2. There is a ‘Cosmic Form’, which *grounds* the causal powers of the Power-Atoms, such that their motion satisfies Born’s distribution.
3. There is a Cosmic Substance composed of Power-Atoms and the Cosmic Form, which has the power to choreograph the Power-Atoms’ trajectories.
4. The Power-Atoms are the substrate of all physical change; it is the distances between them that change.

In what follows, I shall explain how this model grounds a law of nature specified by Bohmian mechanics, which refers to a global configuration of particles, by offering an ontology in which the cosmos is a single substance with an *intrinsic* power to choreograph the trajectories of its particles. I shall begin by explaining the nature and composition of this Cosmic Substance.

### Matter and form

Cosmic Hylomorphism differs from other metaphysical models that adopt a primitive ontology approach to quantum mechanics by positing the existence of an *immaterial* element, over and above the distribution of matter, to explain the temporal development of the matter. In this model, the ‘Power-Atoms’ comprise a substrate of primitive atoms with powers to change their velocities, by changing the distance relations in which they stand. These causal powers are stimulated by the spatial configuration of the Power-Atoms, which is explicated in terms of their distance relations (Suárez 2015). However, the powers of the Power-Atoms are not essential to the Power-Atoms; rather, they are accidental features that change with time. What is essential to the Power-Atoms is their *potential* to bear different powers to change their velocities. Nor are the powers of the Power-Atoms intrinsic to the Power-Atoms; rather, they are extrinsic features of the Power-Atoms that depend on a non-causal interaction with another entity. According to the Cosmic Hylomorphist, the actual powers of the Power-Atoms are *metaphysically grounded* in a ‘Cosmic Form’.

I shall consider the Cosmic Form to be a simple and fundamental particular with the power to manifest a cosmic process (cf. Koons (2018)). It is not *located* in physical space, along with the Power-Atoms, since it does not stand in any distance relations, nor is it the *efficient cause* of their motion, since they have their own causal powers to bring about change. Rather, the Cosmic Form brings about change *indirectly*, in the course of manifesting a cosmic process, by *grounding* the powers of the Power-Atoms at each moment of time. In so doing, I shall argue, the Cosmic Form *unites* itself to all of the Power-Atoms to compose a single substance with an intrinsic power to choreograph the trajectories of the particles through physical space, rather than existing in a transcendent realm of its own. It is the Cosmic Form that explains the diachronic and transworld sameness of the collective power of the cosmic whole.

This model deploys the notion of grounding championed by Schaffer (2009, 2010). I consider the relation of metaphysical grounding to be distinct from any causal relation, such as the causal relation that may exist between one configuration of particles and another. The notion of causal determination does not capture the concept of grounding, since one thing might be causally determined by another thing, yet still have some reality over and above that which causally determines it. In this model, however, the powers of the Power-Atoms depend solely upon the Power-Atoms and the Cosmic Form for their being at each moment in time. Likewise, the concept of supervenience is distinct from the concept of metaphysical grounding, because grounding is a hyper-intensional phenomenon. A modal dependence relation like supervenience, however its formal properties may be construed, cannot capture the idea that the supervenient entities exist ‘in virtue of’ – and are metaphysically explained by – what they supervene on. In this model, the powers of the Power-Atoms are changing features of nature that both depend upon and are explained by a permanent feature of nature; namely, the Cosmic Form, which does not itself depend upon the powers that it confers. Finally, the notion of ontological dependence is not specific enough for the purpose of this model, since *x* may ontologically depend upon *y* without *y* being ontologically *prior* to *x*, whilst the Cosmic Form is ontologically prior to the powers of the Power-Atoms.

This model draws metaphysical inspiration from Aristotle’s hylomorphic account of substance, although it is not identical to Aristotle’s doctrine. For Aristotle, as he has commonly been understood, the substantial form of a substance determines its matter, conferring upon the substance its intrinsic nature and explaining the unity of the substance as a whole, whilst matter is a continuum of potentiality which is to be determined by form. There have been a number of recent attempts to rehabilitate Aristotle’s doctrine of hylomorphism in contemporary metaphysics,^3^ including Morganti’s recent proposal of an ‘amorphic’ version of hylomorphism in the metaphysics of quantum mechanics, which seeks to explain entanglement in terms of a structure of mutual dependencies between physical entities (Morganti 2019).^4^ I shall be employing Koons’ interpretation in terms of metaphysical grounding instead (Koons 2018; 2014). In my metaphysical model, the Power-Atoms are analogous to the Aristotelian concept of matter, even though they are metaphysically discrete. By supplying a persisting substrate with the potential to bear causal powers, the Power-Atoms serve as the *material cause* of the cosmic whole. Likewise, in my model, the Cosmic Form is analogous to the Aristotelian concept of substantial form, although there is only a single form. By grounding the physical powers of the Power-Atoms, the Cosmic Form acts as the *formal cause* of the Cosmic Substance.

### The cosmic substance

Yet how does the unique element of Cosmic Form *unite* itself to the plurality of Power-Atoms to compose a single substance? Or, as E.J. Lowe put the question, in his criticisms of Aristotle’s doctrine of hylomorphism: how are we to ‘justify the judgment that a new concrete object – an “addition of being” – really has been brought into existence, rather than some previously existing things merely being rearranged’ (Lowe 2012, p.236)?

There have been several suggestions among contemporary metaphysicians for how to modify or ‘recondition’ Aristotle’s doctrine of hylomorphism, in order to explain how a form can be the principle of unity of a substance, but I do not propose to adopt any of them. For instance, I do not think a new concrete object is generated simply by adding a special power to unite distinct physical elements (as in Rea (2011)). Such a solution is *ad hoc* and fails to explain in what way this plurality of physical elements is united (Marmodoro 2013). One might be tempted instead to posit some kind of special structure, instantiated by the physical elements, which glues all of them together (as in Jaworski (2016)). However, a structure in which each element is merely causally connected to every other element might still be counted in many ways, according to our explanatory interests, and as such lacks the metaphysical unity of a substance (Marmodoro 2018; Skrzypek 2017).

This problem may be brought into focus by considering Aristotle’s famous syllable regress [Metaphysics VII 17, 1041b, 25–31]. As Aristotle pointed out, a syllable like ‘BA’ is something over and above the letters ‘B’ and ‘A’ of which it is constituted, but merely adding another element would not unify them into a single syllable. Rather, the syllable would then be composed of three elements, which would themselves have to be unified. Likewise, the flesh of an organism is not an aggregate of its basic elements (for Aristotle, ‘earth’ and ‘fire’), since it has causal powers over and above its constituents, and simply adding another element of the same ontological standing would not unify these elements into flesh. What we must add is the *unifying principle* that makes the letter to be a syllable, or the elements to be flesh.

According to Marmodoro, such a principle should not be conceived as an *entity* of any sort, in order to avoid falling into the regress, but as an *act* of conceptual individuation, in which the ultimate constituents of reality are ‘re-identified’ within a whole according to our explanatory interests (Marmodoro 2018). Marmodoro objects to a ‘plurality hylomorphism’ (to use Peterson’s terminology (Peterson 2018)), in which the matter and form of a substance are metaphysical parts, and endorses an ‘anti-plurality hylomorphism’, in which a substance’s matter is not numerically distinct from its form. I suggest, on the contrary, that we can understand how a Cosmic Form that is distinct from the Power-Atoms may unite itself to the Power-Atoms to compose a single substance, without falling into the regress, if this power is conceived as an element of a different ontological standing which *grounds the physical nature* of the composite.

We might get a handle on this solution by considering an analogy suggested by Koons.^5^ Suppose I imagine a 3-4-5 Pythagorean triple. The imagined triangle, if you will, is a compound containing four entities: my act of imagination, and the three lines of the triangle, which are each of distinct lengths. On the one hand, there is clearly a difference between the unique way in which my act of imagination is part of the imagined triangle, and the way in which each of the distinct objects imagined (the lines) are parts. On the other hand, all four of these elements are distinct and disjoint parts of a single whole. The two sorts of elements composing this whole – namely, my act of imagination, and the three different lines – are not of the same ontological standing, since my act of imagination explains why the lines of the triangle exists in the first place. Analogously, I propose that the Cosmic Form is united with the Power-Atoms by being the *formal cause* of the physical whole, which confers upon the matter of the cosmos a single essence or permanent physical nature.

As Anne Peterson points out, objections to plurality hylomorphism, such as those of Marmodoro, contain unstated Quinean assumptions about the univocal nature of being and unity, whereas for Aristotle ‘there is no such thing as just being or just unity; these terms are equivocal’ (Peterson 2018, p.3). If there is no unity that is not ‘unity under some category of being’, the only way to ignite a metaphysical conflict between unity and plurality within a hylomorphic composite is to ‘undermine unity under the category of being at hand’ [p.6]. In this case, the relevant category is that of a *physical substance*, and hence Marmodoro’s objection to plurality hylomorphism reduces to the objection that a unified substance cannot be composed of other substances.

However, Aristotle deploys his distinction between actuality and potentiality to distinguish matter and form from substance: matter is only *potentiality* for actuality, not the substance itself, whereas form ‘is related to a substance as the *actuality* of that substance – its essence, or that which makes it what it is’ [p.8].^6^ Likewise, I claim that Cosmic Hylomorphism is able to resist anti-pluralist objections. First, in drawing upon Aristotle’s doctrine of hylomorphism for metaphysical inspiration, the Cosmic Hylomorphist is consistent in rejecting a univocal notion of unity in favour of Aristotle’s ‘equivocal’ notion instead. Secondly, the Power-Atoms and the Cosmic Form are analogous to Aristotle’s concepts of matter and form in the relevant respects. Neither of these elements is a physical substance. The Power-Atoms are not physical substances because they lack causal powers apart from the Cosmic Form. The Cosmic Form is not a physical substance because it only acts indirectly in the world, conferring a physical nature upon the Power-Atoms and thereby transforming them into the integral parts of a single physical whole.

Marmodoro’s brand of anti-plurality hylomorphism also contains assumptions about the physical world that are difficult to square with quantum mechanics. According to Marmodoro, the world consists of basic ‘power-tropes’, like spin and charge, which are supposedly the properties of particles picked out by our best physics. A hylomorphic substance can only be generated if these power-tropes are, so to speak, dissolved into a unity. This is a subjective transformation affected by ‘a holistic re-individuation of the constituents of the structure, in accordance with either sortal or mass individuation principles’ (Marmodoro 2018, p.66). Yet the phenomenon of quantum entanglement gives us good reason to doubt that quantal properties such as spin are intrinsic properties of particles (Esfeld 2017; Simpson 2017). Furthermore, experimental considerations involving interference phenomena suggest that classical properties, like charge, are best understood on the level of the wave function (Brown et al. 1995, 1996). For Cosmic Hylomorphists, however, there are *no* basic microphysical power-tropes that have to be re-individuated to generate a powerful whole. The physical powers of particles are not intrinsic to any of the particles, and they are not fundamental entities which exist without further explanation, but are grounded in the Cosmic Form.

A metaphysical structure of entities in which one element grounds the physical nature of all the other elements has different properties from a causal structure of entities which each have their own physical natures. A causal structure of physical entities may exhibit novel behaviour, but it is still an aggregate in which every element is causally related to every other element, which can be counted in different ways. However, the structure within which the Cosmic Form and the Power-Atoms are united is not a causal structure. Rather, the Cosmic Form is uniquely and asymmetrically related to all of the Power-Atoms by being the *metaphysical ground* of their causal powers. The Cosmic Form thus serves as the principle of unity of the cosmic whole, by conferring upon the cosmos a single physical nature, but it is of a different ontological standing than the physical parts that it unites. It is an entity in its own right, and an immaterial element of the fundamental ontology.^7^ We might classify it as a *metaphysical power*, or as a *principle of physical being*.

### Laws and powers

How is this Cosmic Form related to the wave function? In a primitive ontology approach to quantum mechanics, the wave function enters the account through the nomological role that it plays in specifying the dynamics of the primitive distribution of matter. Cosmic Hylomorphism spells out its nomological role in two steps: first, by identifying the Cosmic Form as the truthmaker for the lawfulness of the Schrödinger equation; secondly, by identifying the Power-Atoms with the particles to which the Bohmian equation of motion refers.

Let’s begin by considering the lawfulness of the Schrödinger equation, which governs the quantum dynamics of the wave function.^8^ I consider a law to be a general explanatory principle that supports counterfactuals. The relevant conception of lawhood in this case, however, is neither the Mill-Ramsey-Lewis account of laws, nor the Armstrong-Dretske-Tooley account. Matarese has argued that the Mill-Ramsey-Lewis account cannot supply a primitive ontology approach to quantum mechanics with a truthmaker for the lawfulness of its laws that exists independently of our preferences and practices: this combination is susceptible to the ‘problem of immanent comparisons’ (Matarese 2018). The Armstrong-Dretske-Tooley account is also incompatible with a primitive ontology approach to quantum mechanics, because it conceives laws as unchanging relations that necessitate regularities between universal properties. This account cannot accommodate a law of motion that includes a wave function that changes with time, if the wave function is not part of the primitive ontology, since the properties of the wave function are not fundamental physical properties, and thus are ineligible to stand in necessitation relations.

Cosmic Hylomorphism, however, adopts an Aristotelian-essentialist account of laws, in which laws are real but *grounded* in powers (Bird 2007, chp. 9).^9^ For Humeans like Esfeld, the laws are fixed ‘at the end of the world’ by a mosaic of local matters of fact; there is no part of that mosaic that fixes what is physically possible for any other part. For powerists, by contrast, physical modality is not grounded in anything that is non-modal, but in nature’s powers to bring about change by natural necessity. Cosmic Hylomorphism also expands the category of laws to include both *causal* and *metaphysical* laws. Following Schaffer, I take metaphysical explanation to be ‘backed by grounding relations’ and distinct from causal explanation (Schaffer 2017, p.303). Just as causal explanation requires laws of nature to connect causes to effect, for the purpose of unifying natural phenomena and making them intelligible, so metaphysical explanation requires laws to connect grounds to what is grounded, for the purpose of metaphysically unifying that which is grounded by that which is fundamental. Unlike Schaffer, I take causal laws to depend upon powers.

A commitment to an ontology of powers embeds causal explanations within an irreducibly ‘teleological’ context, since powers are *directed* towards their manifestations. Nonetheless, powers are empirically specifiable because their manifestations have nomological profiles: the causal powers of the Power-Atoms, for instance, co-manifest according to the Bohmian law of motion. However, the Bohmian law of motion depends on the wave function of quantum mechanics, which is subject to change according to the Schrödinger equation. According to Bohmians like Dürr and Goldstein, the world at time *t* is characterised by the Bohmian tuple, ({**Q**_*i*_(*t*)},*ψ*(*t*)), which consists of both the global particle configuration **Q**_*i*_(*t*) *and* the universal wave function *ψ*(*t*). What, then, is the ontological status of the wave function?

For Cosmic Hylomorphists, who adopt the primitive ontology approach to quantum mechanics, the wave function is not a physical entity that causally interacts with the Bohmian particles, nor is it a structural entity which they somehow instantiate. Whilst the initial configuration, **Q**_*i*_(*t* = 0), is an *accident* of the Cosmic Substance that could have been otherwise, the initial (or final) value of the wave function, *ψ*(*t* = 0), is an *essential* property of the Cosmic Substance. In this model, the wave function carves a space of possible worlds for this self-same Cosmic Substance, according to the Schrödinger equation and the Bohmian law of motion, in which initial positions of the particle configuration are paired with potential trajectories through physical space:

$$ \langle \{\mathbf{Q}_{i}(0)\}, \{\mathbf{Q}_{i}(t)\} \rangle , \quad \langle \{\mathbf{R}_{i}(0)\} , \{\mathbf{R}_{i}(t)\} \rangle, \quad \langle \{\mathbf{S}_{i}(0)\}, \{\mathbf{S}_{i}(t)\} \rangle, \quad \textellipsis $$

The metaphysical law that connects initial positions to potential trajectories is not something over and above nature’s basic constituents, as in Maudlin’s primitivist conception of laws (Maudlin 2007), nor does it merely describe a regularity observed in physical space, as in Esfeld’s Super-Humean metaphysics (Esfeld and Deckert 2017). Rather, I propose that this law expresses the metaphysical power of the Cosmic Form to manifest a cosmic process, which can be usefully characterised by the universal wave function. The Cosmic Form manifests this process by means of a structure of grounding relations that unite the Power-Atoms to the Cosmic Form, in which it grounds the powers of the Power-Atoms at each moment.

This metaphysical structure is irreducible to any structural property of the particles in *physical* space, since the wave function does not supervene upon any arrangement of the particles in physical space. Nonetheless, it can be represented in the system’s *configuration* space as a series of wave fronts, which push the particles along trajectories that are perpendicular to surfaces of constant phase, although many of these potential trajectories are not actualised. According to this model, the world would still be a *Bohmian* cosmos if it had a different wave function, but it would not be the self-same *Cosmic Substance* without these characteristic wavefronts in the system’s configuration space. In this way, the wave function represents the ontological dependence of the powers of the Power-Atoms on the Cosmic Form at each moment of time,^10^ and in different possible worlds, without itself being an element of the primitive ontology. As Kim observes: ‘The ontological contribution of dependence relations lies exactly in this fact: they reduce the number of independent events, states, facts, and properties we need to recognize’ (Kim 1994, p.68).

Cosmic Hylomorphism thus rejects both the Humean characterisation of the nomological role of the wave function in terms of a ‘best systems’ account of laws, in which the wave function supervenes upon the total configuration of particles for all time, and standard powerist characterisations of the nomological role of the wave function in terms of a structural power, in which this power is extrinsic to the particle configuration. In this alternative metaphysical model, the Schrödinger equation represents a metaphysical law, which is grounded in the Cosmic Form, and the Bohmian law of motion represents a causal law, which expresses the intrinsic power of the Cosmic Substance to choreograph the trajectories of its particles through physical space.

### Powers and properties

So how do the physical properties of the objects of scientific inquiry fit into this picture? In a primitive ontology approach to quantum mechanics, the *matter* that composes physical objects, such as the macroscopic instruments upon which scientists depend, lacks any intrinsic physical properties. Properties such as mass and charge enter into the metaphysical account through the dynamical role they play in describing regularities in the distribution of matter.

Nonetheless, although Cosmic Hylomorphism adopts the primitive ontology approach to quantum mechanics, the Cosmic Hylomorphist rejects the Super-Humean characterisation of physical properties as being nothing but useful and informative ways of representing change in the distribution of matter (Esfeld and Deckert 2017, p.7). The Cosmic Substance, which is a hylomorphic composite of both matter *and* form, is a physical substance which has intrinsic physical properties, such as the power to choreograph the trajectories of its particles. The lesson of quantum mechanics for contemporary metaphysics, from this metaphysical standpoint, is not captured by the radically revisionary claim that there are no intrinsic properties whatsoever, as Esfeld contends (Esfeld 2014b), or that there are no fundamental physical objects, as French and Ladyman seem to suppose (French and Ladyman 2003). Rather, the lesson of quantum mechanics can be heard as a clarion call to return to a *hylomorphic* conception of physical properties as causal powers which are intrinsic to substances composed of matter and form.

The Cosmic Hylomorphist adopts this hylomorphic conception of physical properties in the ‘staunch’ sense that has been put forward by Koons, in which the matter and form of a substance are part of the fundamental ontology (Koons 2014). In my model, material particles do not have intrinsic physical properties, like mass or charge, nor do they collectively instantiate a structural power, as a brute matter of fact. Rather, there is a Cosmic Form which *grounds* the causal powers of the Power-Atoms, and the Power-Atoms then manifest those causal powers. Since the Cosmic Form unites itself to the Power-Atoms to compose a single substance, we may think of the trajectories of the Power-Atoms as being ‘choreographed’ by an *intrinsic power* of the Cosmic Substance, in accordance with the Bohmian law of motion. It is this hylomorphic substance, rather than matter *per se*, which is the subject of intrinsic physical properties.

### Limitations of the model

According to the version of Cosmic Hylomorphism that I have expounded in this paper, the Schrödinger equation represents a metaphysical law, which is grounded in the Cosmic Form, and the Bohmian law of motion represents a causal law, which expresses an intrinsic power of the Cosmic Substance. Of course, this metaphysical model comes with certain limitations.

In the first place, the physical setup is only provisional. We know that Bohmian Mechanics – in the standard, non-relativistic form in which it is usually presented – is just an effective theory that is only valid at certain length scales. Relativistic extensions of Bohmian mechanics have been proposed, however, which replace the Schrödinger equation with a different quantum dynamics, and modify the equation of motion (Dürr et al. 2014). Likewise, Bohmian representations of quantum field theory – in terms of a single particle configuration – are also being explored (Deckert et al. 2019), and Bohmians are hopeful of offering particle representations of any future theory of quantum gravity (Vassallo and Esfeld 2014; Esfeld 2019). From a general hylomorphic perspective, I suggest, these physical theories do not demand a different metaphysical model, but offer different *physical* characterisations of the causal power that is exercised by the Cosmic Substance.

In the second place, this model shares the explanatory limitations of the primitive ontology approach to quantum mechanics, which assumes that the whole world can be physically characterised as having a quantum state defined in a single Hilbert space. I am doubtful that this is the case. As Laura Ruetsche has argued, in order to fulfill its explanatory virtues, quantum mechanics must admit multiple interpretations that adopt different Hilbert-space representations (Ruetsche 2011): it seems that *unitarily inequivalent* representations are required in order to capture all of the phenomena that quantum theories are supposed to explain (Ruetsche 2003). I do not seek to defend the primitive ontology approach to quantum mechanics from these kinds of criticisms in this paper, nor do I wish to endorse Bohmian mechanics as the best interpretation of quantum mechanics. I am content rather to use this approach as a way of providing a ‘toy model’ in which to explore the debate between powerists and Humeans – one which can accommodate the puzzling phenomenon of quantum entanglement.

## Advantages of cosmic hylomorphism

My primary concern in this paper has been to offer a powerist explication of the nomological role of the wave function in the primitive ontology approach to quantum mechanics. Powerists complain that Humean laws do not explain behaviour, since they are grounded in the instances that they are supposed to explain. A law that is grounded in the power of a substance may be explanatory, however, if the substance produces the law-like behaviour by exercising an intrinsic power. Yet how would a cosmic power have to operate to explain the phenomenon of quantum entanglement, which Schrödinger described as both ‘the characteristic trait of quantum mechanics’ and a concept ‘that enforces its entire departure from classical lines of thought’ (Schrödinger 1935)? This is another question a Bohmian powerist will have to address.

Let us distinguish between a *weak* power, which operates as a modal, nomic structure that constrains the motion of the Bohmian particles in some way, from a *strong* power, which produces the motions of the Bohmian particles (either directly, or by conferring causal powers on the particles, which in turn produce the motion).^11^ Morganti’s suggestion that we should understand entanglement correlations in terms of some structure of mutual dependence between particles which have physical dispositions, for instance, seems to be ambiguous between these two conceptions of a global power (Morganti 2019). It seems that powerists require a strong type of power, however, in order to secure the kind of explanation of the motion of Bohmian particles that they find lacking in Super-Humeanism. Yet Super-Humeans may object that a strong power would have to operate in an unlawful or implausible or extravagant way, in order to explain the existence of quantum-entanglement correlations. In that case, the explanatory advantage that powerists seek to gain over Humeans may not be possible in the case of quantum phenomena, or at least in the context of a primitive ontology approach to quantum mechanics.

### Non-local EPR correlations

The phenomenon I wish to discuss concerns systems of particles in which the outcomes of measurements are correlated in such a way that the quantum state describing the system cannot be decomposed into the product of separate states associated with each particle. For example, in the famous EPR experiment involving two particles, originally proposed as a thought-experiment by Albert Einstein and his associates in 1935 (Einstein et al. 1935), one particle is constrained to be ‘spin-up’ $\left | \uparrow \right \rangle $ when another is measured to be ‘spin-down’ $\left | \downarrow \right \rangle $, and vice versa, however far apart the two particles are spatially separated. The system in this case is in a superposition described by the famous singlet state:

1 $$ \left| \psi \right\rangle = \frac{1}{\sqrt{2}} (\left| \uparrow \right\rangle_{1} \left| \downarrow \right\rangle_{2} - \left| \downarrow \right\rangle_{1} \left| \uparrow \right\rangle_{2} ). $$

When a system is in the singlet state, there is a probability of 1/2 that we will observe particle 1 to be ‘spin up’, and particle 2 to be ‘spin down’; there is also a probability of 1/2 that we will observe particle 1 to be ‘spin down’, and particle 2 to be ‘spin up’. There are no other physically possible outcomes.

Following Esfeld’s discussion in (Esfeld 2017), I shall briefly review how this entangled behaviour arises. Suppose two particles emitted from a common source fly off in opposite directions, and two experimenters (traditionally, ‘Alice’ and ‘Bob’) are suitably positioned to measure certain physical observables associated with each particle, once the two particles are sufficiently separated. In his ground-breaking discussion of quantum entanglement, the physicist John Bell formulated a theorem drawing a radical distinction between the local behaviour explained by classical physics and the non-local behaviour of systems governed by quantum physics (Bell 1964). In order to describe its implications more precisely, it will be helpful to introduce some notation.

Let *ϕ*_*A*_ specify the configuration of Alice’s apparatus, and *A* the outcome of her experiment; likewise, *ϕ*_*B*_ for Bob’s apparatus, and *B* for his outcome. Let *λ* denote whatever in the past may have influenced the behaviour of the system that is being measured. In our example, *λ* includes the physical state of the two-particle system, prior to measurement. The measuring apparatus in each case is a Stern-Gerlach device, in which a pointer has the possibility of being deflected up or down, and the configuration parameters are the two angles of polarisation of each device, *ϕ*_*A*_ and *ϕ*_*B*_. These parameters can be set at an appropriate angle for measuring vertical spin (that is, ‘spin-up’, or ‘spin-down’), but can also be adjusted separately to produce a range of measurement outcomes. According to Bell, the principle of locality requires:

2 $$ \begin{array}{@{}rcl@{}} P_{\phi_{a},\phi_{b}} (A | B, \lambda) &= P_{\phi_{a}} (A | \lambda), \end{array} $$

3 $$ \begin{array}{@{}rcl@{}} P_{\phi_{a},\phi_{b}} (B | A, \lambda) &= P_{\phi_{b}} (B| \lambda). \end{array} $$

This formalisation can be read as follows: according to the principle of locality, the probabilities for Alice obtaining outcome *A* can be fixed by conditionalising on the configuration of her apparatus *ϕ*_*A*_, and whatever in the past influenced its behaviour *λ*, such as the local properties of the particle she measures. Significantly, conditionalising on the configuration of Bob’s apparatus, in a world in which locality holds, does not change the probabilities for Alice’s outcome. This is also the case for Bob’s outcome with respect to Alice’s apparatus.

Bell’s theorem demonstrates that the principle of locality is violated by the phenomenon of quantum entanglement. In this way, Bell problematised the correlations between spatially separated systems that are quantum-entangled for any physical theory in which the prospect for transmitting a signal that causally influences a physical system is limited by the speed of light. According to quantum mechanics, the probabilities for obtaining a measurement outcome in one part of the experiment depend on the outcome obtained in the other part of the experiment, in spite of the fact that the two measurement events are represented as ‘spacelike separated’ in the theory of special relativity.

To visualise the limits on causal signalling imposed by the theory of relativity, the mathematician Minkowski suggested imagining a flash of light, confined to a two-dimensional plane, which spreads out in a circle from an event *E* at some time *t* (Minkowski 1908). If we graph the growing circle using time as the vertical axis, we obtain a ‘light-cone’ for event *E* that extends to include any past event *t*^′^ < *t* in which a signal could have been sent which would have time to reach *E* and causally influence this event. However, any event which falls outside of the light-cone of *E* is ‘spacelike separated’ from *E*, and cannot be causally related to *E* by any mechanism. In other words: the physical (efficient) cause of a physical effect must lie within its past light-cone.

Since the measurement events in the two wings of the EPR experiment corresponding to *A* and *B* do not belong within each other’s past light-cones, they are space-like separated. However, applying the ‘classical’ assumption that the behaviour of the particles can be explained by a local mechanism implies one set of measurement statistics, whereas quantum mechanics predicts another. Significantly, in the case of the EPR experiment, quantum mechanics predicts that the measurement statistics will depend on the relative angle between the two devices, *ϕ*_*A*_ − *ϕ*_*B*_. This fact is something that neither particle, considered separately, could possibly ‘know’ by means of any subluminal mechanism. Whilst the EPR experiment was originally intended as a *reductio ad absurdum* of quantum mechanics by Einstein, subsequent experiments – in particular, those of Aspect in the 1980s (Aspect et al. 1982) – are now widely regarded as having confirmed the statistics predicted by quantum mechanics and established non-locality as an empirical fact in the following sense:

What happens at point *P* in space and time depends not only upon what exists in the past light-cone of this event, but also upon what happens outside of its light-cone, at points in space separated from *P* by a space-like interval.^12^

This behaviour is not merely a peculiarity of early pioneer quantum mechanics: it remains a puzzling feature of more sophisticated quantum field theories deployed by the Standard Model of particle physics, and is likely to remain a feature of any theory of quantum gravity. The problem posed by the phenomenon of quantum entanglement to philosophers is the challenge of explaining the non-local measurement outcomes of quantum experiments given the ban on superluminal signalling that applies to mechanisms in modern physics.

### Reality versus appearance

One might try to explain away the *appearance* of non-local dependencies by looking for loopholes in Bell’s theorem that can be used to account for non-local measurement outcomes. For example, some have claimed that the settings of the measuring devices, *ϕ*_*A*_ and *ϕ*_*B*_, are not in fact independent of whatever in the past may have influenced the behaviour of the system that is being measured; namely, *λ*. There are two possibilities: either *λ* must be influenced by the measurement settings, or the measurement settings influenced by *λ*.

To advance the first possibility would be to make an exotic appeal to time travel, requiring that the measurement settings of our devices (or the outcomes, which depend on the settings) somehow causally influence the state of the system being measured retroactively. To advance the second would be to posit a common cause of the measurement outcomes somewhere in the intersection of their past light-cones, which accounts for both the experimental outcomes, *A* and *B*, *and* the settings of the measuring devices, *ϕ*_*A*_ and *ϕ*_*B*_.

Whilst the first logical possibility stretches credulity, and appears to invite the possibility of paradox, the plausibility of the second has sometimes been defended by the claim that the negation of the independence of these variables logically follows from the truth of *determinism*, and that to believe otherwise would be to attribute to scientists who construct experiments a form of ‘free will’ that is incompatible with determinism. However, this is a *non sequitur*, since the independence of these variables is logically consistent with a deterministic universe where that which determines *λ* and that which determines *A* and *B* are distinct in the initial state of the universe. In order to exploit this loophole in Bell’s theorem, we would have to endorse a form of *superdeterminism*, sacrificing a general assumption of scientific practice: namely, that in making a measurement, the settings of the device used to make that measurement should be independent of the prior state of the measured system.^13^

Bohmian mechanics takes the non-locality of quantum mechanics seriously, providing an account of the EPR experiment in terms of both the wave function *and* particle positions, where the initial conditions of the particles and of the wave function are specified separately. According to Bohmians, quantum spin is explained by the wave function of a particle and its initial position. (The wave function in this case is the *conditional* wave function of the subsystem, not the *universal* wave function of the global configuration, of which this particle is a part.) Suppose we direct a spin 1/2 particle at the magnets of a Stern-Gerlach device, such that its wave function splits into ‘up’ and ‘down’ wavepackets. The way in which the particle travels within the apparatus – its ‘choice’, let’s say, to travel up rather than down – is determined by its initial position in relation to the magnets. If this particle is entangled with another particle, however, that choice changes the wave function of the other particle, guaranteeing that it will go down when it is measured. In effect, the wave function of the second particle has been forced to evolve non-locally in three-dimensional space, with the local behaviour of particle 1 nomically determining the wave function of particle 2. Consequently, after the measurement of particle 1, the ‘spin’ of particle 2 – and hence its trajectory – is no longer a function of its position in relation to the magnets that are used to measure it.

### Formal versus efficient causation

There is more than one way to think about this non-local dependency relation, however, according to how we conceive the fundamental ontology. For Super-Humeans, the particles are matter points, which are nothing over and above the distance relations in which they stand, and the non-local correlations are a brute fact about how they are distributed in space and time. For Cosmic Hylomorphists, the particles are Power-Atoms, whose causal powers are grounded in a Cosmic Form, and the non-local correlations must be related to the operation of the Cosmic Form which is manifesting the cosmic process.

However, Super-Humeans may complain that appealing to a strong power to explain the phenomenon of quantum entanglement implicates powerists in an implausible conception of how this power would have to operate: in order to explain the non-local correlations of the EPR experiment we must suppose there to be something in the physical world that is simultaneously coordinating the motions of all the particles from moment to moment, such that they are compelled to follow the trajectories determined by the Bohmian law. Yet it seems incredible to suppose that there is a *physical mechanism* with such organisational power, which superluminally choreographs the trajectories of all the particles without transmitting any detectable signals, and a causal explanation of the non-local correlations runs the risk of superdeterminism. But this is not the Cosmic Hylomorphist’s conception of how the Cosmic Substance choreographs the trajectories of the particles.

Suppose we say that particles 1 and 2 had powers to travel up or down within the Stern-Gerlach apparatus, and that the power of particle 1 to travel up was stimulated by its initial position *x*_1_. Had particle 1 occupied an initial position of *y*_1_, however, it would have manifested the opposite power. In that case, it appears that the corresponding powers of particle 2 in the EPR experiment must change when particle 1 is measured, because certain counterfactuals with respect to particle 2 have changed in their truth values. For instance, before a measurement of particle 1 takes place, it is true of particle 2 that its spin is a function of its initial position. Suppose that particle 2 is ‘spin-down’ and deflects downwards, if it is in the initial position *x*_2_ in relation to the magnets. Because this trajectory is determined by its initial position *x*_2_, it is true that if particle 2 had occupied the initial position *y*_2_ it would have been ‘spin-up’ and deflected upwards instead. Yet this counterfactual no longer holds following measurement. After the measurement of particle 1, we know that particle 2 will be ‘spin-down’ and deflect downwards, independently of its initial position. The powers of particle 2 appear to have changed.

Nonetheless, this is consistent with the picture of nature presented by Cosmic Hylomorphism, since the Cosmic Form changes the powers of particle 2 after particle 1 is observed to be ‘spin-up’. According to Bohmian mechanics, a system of particles that is under observation cannot be a closed system, but must be a subsystem of a larger closed system. This larger system is ultimately nothing less the entire cosmos itself, whose particles are ‘guided’ by the universal wave function. According to the Cosmic Hylomorphist, the Power-Atoms, which correspond to the Bohmian particles, are caught up within a cosmic process, which is characterised by the universal wave function, and their causal powers are continually changing in a such a way that they satisfy the Bohmian law of motion, which depends on the universal wave function.^14^ The initial configuration of the particle configuration is not determined, however, but is an accidental property of the Cosmic Substance, so they can follow different trajectories in different possible worlds. Superdeterminism is thus avoided.

Does this instantaneous change unlawfully violate the superluminal ban on signalling? I think not. For both the Super-Humean and the Cosmic Hylomorphist, the Lorentzian structure of spacetime that imposes the superluminal ban on signalling between physical systems is an emergent fact about the properties of local sub-systems, rather than a fundamental law.^15^ Moreover, the Cosmic Form which unites itself to the Power-Atoms, by grounding their causal powers, is the *formal cause* of the unified physical substance, rather than an efficient cause of an extrinsic organisation that is imposed upon a set of physical particles.^16^ The Power-Atoms, thus ‘empowered’, are at once united as integral parts of a single physical whole, rather than separate physical entities that must be micro-managed according to a physical mechanism. Their powers and positions constitute the accidental form of this physical substance.

I submit that an instantaneous ‘formal’ cause of the non-local correlations associated with the phenomenon of quantum entanglement is not unlawful in the way that an instantaneous ‘efficient’ cause seems to be: it is efficient causes, not formal causes, which must abide by the superluminal ban. Likewise, this model is not as implausible as a cosmic conspiracy among the particles: we have experience of physical parts being co-opted by the goals of an organic entity. Nor is this proposal as ontologically extravagant as the claim that every particle must have an infinite number of basic powers, which they exercise in response to different configurations (Suárez 2015): Cosmic Hylomorphism admits only one fundamental power and only one fundamental substance.

Such a notion of physicality, however, in which a physical entity can be analysed in terms of the metaphysical concepts of matter and form, requires us to divorce the metaphysics of physics from the philosophy of materialism. A second lesson of quantum mechanics, from this hylomorphic standpoint, is that nature does not wear all of her properties on her sleeves. Whatever the aspiration of early scientists like Gassendi, who shunned ‘occult’ natures and scorned Aristotelian forms of explanation, it seems we can only get so far in cashing out the nature of things in terms of empirical properties of their matter whose values can be determined directly in an experiment. The nature of the cosmos, according to the Cosmic Hylomorphist, does not reduce to the properties of its matter, any more than a wave function may be said to be an intrinsic property of a physical particle or to supervene upon a particle configuration. There is something ‘hidden’ in nature which can only be uncovered through a process of metaphysical abstraction. For Cosmic Hylomorphists, what is hidden is the *telos* of the Cosmic Form, which grounds the physical nature of the cosmos. Yet the operation of the Cosmic Form is not without empirical evidence. The non-local correlations of quantum mechanics are its signature.

## Concluding remarks

I began this discussion by noting two ways of spelling out the nomological role of the wave function in the primitive ontology approach to quantum mechanics: by appealing to a Humean notion of laws, in which laws describe regularities, or to some form of powerism, in which laws are grounded in powers. In this paper, I have advanced a powerist model which adapts the primitive ontology of Bohmian mechanics put forward by Goldstein et al. (2005a, b): Cosmic Hylomorphism offers a metaphysics of physics in which the primitive ontology consists of Power-Atoms, which have powers to change their velocities, and there is also a Cosmic Form, which grounds their changing causal powers.

By identifying the Power-Atoms with the particles of Bohmian mechanics, and identifying the Cosmic Form as the truthmaker for the lawfulness of the Schrödinger equation, this model supplies an adequate supervenience base for the laws of Bohmian mechanics. By adopting a hylomorphic conception of substance, in which the Power-Atoms and the Cosmic Form compose a single Cosmic Substance, this model is able to offer a powerist ontology in which the cosmos has an *intrinsic* power to choreograph the trajectories of the particles, which is immune to Molière-type objections. It also provides a unique explanation of the non-local correlations associated with the phenomenon of quantum entanglement in terms of Aristotle’s concept of formal causation.

In constructing this metaphysical model for the theory of Bohmian mechanics, I have sought to supply a powerist alternative to Super-Humeanism, which is the most developed primitive ontology approach to Bohmian mechanics that is currently available, and to highlight the shortcomings of less developed powerist models, which fail to offer a clear explanatory advantage. I have also tried to demonstrate how the metaphysics of hylomorphism can be applied to a comparatively simple and well-understood interpretation of quantum mechanics – albeit an interpretation to which I do not subscribe – with a view to extending hylomorphic treatments of this kind to other interpretations.

## Footnotes

1 For textbook studies, see (Holland 1993; Dürr and Teufel 2009; Dürr et al. 2012; Bricmont 2016).

2 For example, those who embrace Armstrong’s necessitation theory of laws.

3 Including book-length accounts of hylomorphism by Koslicki (2008, 2018) and Jaworski (2016).

4 Morganti prefers to conceive form in terms of this structure of ontological dependencies rather than as an element of the fundamental ontology.

5 Personal correspondence.

6 Emphasis added.

7 In scholastic terminology: it exists *ante rem*, and not *post rem*.

8 In quantum cosmology, the wave function is supposed to solve the Wheeler-DeWitt equation. Like Esfeld, however, I shall rely upon the account of spacetime developed by Huggett, treating spacetime as a useful construct for representing the change in the particles’ distance relations, and as having ‘no reality beyond their assumption by the laws’ (Huggett 2006, p.57).

9 See Mumford (2005) for an eliminativist view of laws, in which powers supplant laws.

10 We might think of this abstract form of dependence as being analogous to how the motions of balls on curved surfaces depend upon the geometry of the surface.

11 I should like to thank Michael Esfeld for suggesting this distinction.

12 This formulation is adapted from Esfeld (2017).

13 According to Anton Zeilinger, scientists ‘always implicitly assume the freedom of the experimentalist... This fundamental assumption is essential to doing science. If this were not true, then, I suggest, it would make no sense at all to ask nature questions in an experiment, since then nature could determine what our questions are, and that could guide our questions such that we arrive at a false picture of nature’ (Zeilinger 2010, p.266).

14 The quantum equilibrium hypothesis must be assumed as a brute matter of fact.

15 Super-Humeans rely upon the regularity account of spacetime developed by Huggett, in which the geometric structures deployed in Newtonian mechanics or the theory of Special Relativity are regarded as useful constructs for representing the change in the particles distance relations, and as having ‘no reality beyond their assumption by the laws’ (Huggett 2006, p.57).

16 According to Aquinas, the operation of a substantial form is instantaneous (Aquinas 1272, Par. 70).
